# Hydrogel Formulations Incorporating Drug Nanocrystals Enhance the Therapeutic Effect of Rebamipide in a Hamster Model for Oral Mucositis

**DOI:** 10.3390/pharmaceutics12060532

**Published:** 2020-06-09

**Authors:** Noriaki Nagai, Ryotaro Seiriki, Saori Deguchi, Hiroko Otake, Noriko Hiramatsu, Hiroshi Sasaki, Naoki Yamamoto

**Affiliations:** 1 Faculty of Pharmacy, Kindai University, 3-4-1 Kowakae, Higashi-Osaka, Osaka 577-8502, Japan; 1611610157u@kindai.ac.jp (R.S.); 2045110002h@kindai.ac.jp (S.D.); hotake@phar.kindai.ac.jp (H.O.); 2 Laboratory of Molecularbiology and Histochemistry, Fujita Health University Institute of Joint Research, 1-98 Dengakugakubo, Kutsukake, Toyoake, Aichi 470-1192, Japan; norikoh@fujita-hu.ac.jp; 3 Department of Ophthalmology, Kanazawa Medical University, 1-1 Daigaku, Uchinada, Kahoku, Ishikawa 920-0293, Japan; sasaki-h@k5.dion.ne.jp (H.S.); naokiy@kanazawa-med.ac.jp (N.Y.)

**Keywords:** rebamipide, nanocrystals, oral mucositis, hydrogel, endocytosis

## Abstract

A mouthwash formulation of rebamipide (REB) is commonly used to treat oral mucositis; however, this formulation does not provide sufficient treatment or prevention in cases of serious oral mucositis. To improve treatment, we attempted to design a hydrogel incorporating REB nanocrystals (R-NPs gel). The R-NPs gel was prepared by a bead mill method using carbopol hydrogel, methylcellulose and 2-hydroxypropyl-β-cyclodextrin, and another hydrogel incorporating REB microcrystals (R-MPs gel) was prepared following the same protocol but without the bead mill treatment. The REB particle size in the R-MPs gel was 0.15–25 μm, and while the REB particle size was 50–180 nm in the R-NPs gel. Next, we investigated the therapeutic effect of REB nanocrystals on oral mucositis using a hamster model. Almost all of the REB was released as drug nanocrystals from the R-NPs gel, and the REB content in the cheek pouch of hamsters treated with R-NPs gel was significantly higher than that of hamsters treated with R-MPs gel. Further, treatment with REB hydrogels enhanced the healing of oral wounds in the hamsters. REB accumulation in the cheek pouch of hamsters treated with the R-NPs gel was prevented by an inhibitor of clathrin-dependent endocytosis (CME) (40 μM dynasore). In conclusion, we designed an R-NPs gel and found that REB nanocrystals are taken up by tissues through CME, where they provide a persistent effect resulting in an enhancement of oral wound healing.

## 1. Introduction

Pain caused by serious oral mucositis affects food intake, nutrition, speaking, and swallowing, can cause life-threatening bacteremia, and ultimately leads to poor quality of life for patients [1,2]. Chemoradiotherapy for patients with head and neck cancer often causes serious oral mucositis, and the severe pain interferes with subsequent treatment and quality of life [3,4,5]. Previous studies have shown that reactive oxygen species cause cell apoptosis, DNA damage, and enhanced production of proinflammatory cytokines [6], and that these factors greatly impact mucositis. Therefore, topical granulocyte macrophage colony stimulating factors, anti-inflammatory agents and mucosal coating agents are widely used as medical therapies for oral mucositis [7]. In addition, Caphosol^®^, MuGard^®^, Mucotrol™, Gelclair^®^, Episil^®^ and Palifermin have surfaced on the market. Caphosol^®^ is a supersaturated calcium phosphate, electrolyte mouth rinse used as artificial saliva, and MuGard^®^ (oral mucoadhesive) causes the formation of a protective coating over oral mucosa. Mucotrol™ is a mixture of herbal agents that is used as an oral gel wafer and includes sorbitol, *Cyamopsis tetragonolobus*, stearic acid, magnesium stearate and aloe. Gelclair^®^ is a viscous gel that is used as a mouthwash and forms a protective film by adhering to the mucosa of the oropharyngeal cavity, helping to provide pain relief in mouth lesions. Episil^®^ is a lipid-based fluid developed for the management and relief of pain associated with oral lesions of various aetiologies such as oral mucositis, a painful side effect of cancer therapies. Palifermin is a truncated human recombinant keratinocyte growth factor produced in *Escherichia coli*. The keratinocyte growth factor stimulates the growth of cells that line the surface of the mouth and intestinal tract. However, serious oral mucositis remains a frequent and critical complication of head and neck cancers [8,9,10].

Rebamipide (REB), 2-(4-chlorobenzoylamino)-3-[2(1H)-quinolinone-4-yl]-propionic acid, was developed by Otsuka Pharmaceutical Co., Ltd. (Tokyo, Japan) for the treatment of gastric ulcers, gastritis, and dry eye syndrome. It was reported that REB scavenges free radicals, exhibits an anti-inflammatory action and improves blood flow [11,12]. In addition, REB increases endogenous prostaglandins E2 and I2, leading to antibacterial effects, mucin secretagogue activity and anti-inflammatory action [13,14]. The efficacy of REB for the treatment of oral mucositis was first reported by Matsuda et al. in 1994, and a resulting mouthwash containing REB has been used as a therapy for mucositis caused by radiotherapy [15], chemotherapy [16] and Behcet’s disease [7]. Furthermore, a pilot randomized controlled trial (RCT) reported that mouthwash containing REB reduces the onset of oral mucositis caused by chemoradiotherapy and radiotherapy [15,17,18]. The various pharmacological effects of REB include suppression of the induction of mucus secretion [19], promotion of endogenous prostaglandin production in the gastric mucosa [20,21], anti-free radical action [12], neutrophil activation [22,23], inhibition of inflammatory reactions [24,25,26] and up-regulation of epidermal growth factor and its receptor [27]. Pharmacokinetic studies using experimental animals have shown that REB acts directly on peptic ulcers and gastritis [28], and that a 4% REB liquid preparation is the optimal concentration for a mouthwash in terms of safety and efficacy profiles [18]. However, sufficient drug efficacy is difficult to obtain since the mouth-washing agent has a short residence time in the oral cavity, and a local concentration of REB on the oral mucosa cannot be maintained via a mouthwash containing liquid REB. We aimed to prepare mucoadhesive formulations, and focused to design the formulations incorporating drug nanocrystals into a hydrogel net. Moreover, we investigated the mucoadhesive properties, drug release and the uptake of REB into the cheek pouch tissue in the REB hydrogel.

It is important to consider the structure of the three-dimensional network in sputum to efficiently deliver REB particles to the oral mucosa. It has been reported that gastrointestinal mucus and cystic fibrosis sputum almost completely block the delivery of particles larger than 500 nm [29,30,31]. In addition, the mucoadhesive properties of a formulation are enhanced by decreasing their particle size, since this increases the relative surface area [32]. Therefore, we hypothesized that nanocrystals with a particle diameter smaller than 200 nm would be a suitable carrier of REB for the treatment of oral mucositis. We previously found that drug nanocrystals were taken up by cells, provided high efficiency, and showed that a hydrogel is suitable as a base for gel formulations, since the drug nanocrystals were easily released from the hydrogel base [33,34,35]. In this study, we attempted to design a hydrogel formulation containing REB nanocrystals smaller than 200 nm by incorporating the drug nanocrystals into a hydrogel net, and to demonstrate the usefulness of these REB nanocrystals as a therapy for oral mucositis.

## 2. Materials and Methods

### 2.1. Animals

Male golden or Syrian hamsters (*Mesocricetus auratus*, weight 98 ± 2.6 g) were purchased from Shimizu Laboratory Supplies Co., Ltd. (Kyoto, Japan). All animal experiments were approved by the animal experimental committee in Kindai University on 1 April 2019 (approval number KAPS-31-016).

### 2.2. Preparation of REB Hydrogel

Hydrogel formulations incorporating REB nanocrystals were prepared according to previous reports [33,34]. REB powder (0.4%; Wako Pure Chemical Industries, Ltd., Osaka, Japan), 0.5% methylcellulose (MC; Shin-Etsu Chemical Co., Ltd., Tokyo, Japan) and 5% 2-hydroxypropyl-β-cyclodextrin (HPβCD; Nihon Shokuhin Kako Co., Ltd., Tokyo, Japan) were added to distilled water containing 0.1 mm zirconia beads, and crushed at 5500 rpm for 1 min by a Micro Smash MS-100R (TOMY SEIKO Co. Ltd., Tokyo, Japan). The mill treatment was repeated 30 times, after which the dispersions were milled at 1500 rpm for 3 h with a Shake Master NEO (Bio-Medical Science Co., Ltd., Tokyo, Japan). The milled dispersions were incorporated into a Carbopol^®^ 934 hydrogel net (Serva, Heidelberg, Germany) and used as hydrogel formulations incorporating REB nanocrystals (R-NPs gel) in this study. Hydrogel formulations incorporating REB microcrystals (R-MPs gel) were prepared by mixing the 0.4% REB powder, 0.5% MC and 5% HPβCD in the distilled water, and incorporated the REB microcrystals into Carbopol^®^ 934 hydrogel net.

### 2.3. Characteristics of REB Hydrogels

The characteristics of the REB hydrogels were analyzed according to previous studies [33,34]. Briefly, the particle size distributions of the R-MPs gels were measured by a SALD-7100 (refractive index, 1.60-0.10i; Shimadzu Corp., Kyoto, Japan), and the particle size distribution and particle number of the R-NPs gel were determined using NANOSIGHT LM10 (viscosity, 1.27 mPa⋅s; QuantumDesign Japan, Tokyo, Japan). The atomic force microscopy (AFM) image of the REB nanocrystals was obtained using a SPM-9700 (Shimadzu Corp., Kyoto, Japan), and the crystal form was determined by a powder X-ray diffraction (XRD) Mini Flex II (Rigaku Co., Tokyo, Japan). The zeta potential of REB was analyzed by a model 502 zeta potential analyzer (Nihon Rufuto Co., Ltd., Tokyo, Japan). REB concentrations were measured on a HPLC LC-20AT system (Shimadzu Corp. Kyoto, Japan) with an Inertsil^®^ ODS-3 column (GL Science Co., Inc., Tokyo, Japan) with detection at 287 nm. Methyl p-hydroxybenzoate was used as an internal standard, and the mobile phase was 50 mM phosphate buffer/acetonitrile (75/25 *v*/*v*) at a flow rate of 0.25 mL/min. The uniformity of the REB in the gels was determined as follows: 0.3 g of REB hydrogel was divided into 10 parts (0.03 g) and dissolved in *N*,*N*-dimethylformamide. The REB contents in the dissolved samples were measured by the HPLC method described above. In this study, the standard deviation (SD) of the REB levels in the 10 hydrogel divisions represents the non-uniformity of REB in the hydrogel.

### 2.4. Permeation Study from REB Hydrogels

Drug release from the REB hydrogels was analyzed according to the previous study using a Franz diffusion cell set on an MF™-MEMBRANE FILTER with a pore size of 220 nm (Merck Millipore, Tokyo, Japan) [33,34,35]. The reservoir chamber of the cell was filled with 12.2 mL of 10 mM phosphate buffer consist of sodium phosphate and potassium phosphate according to the Japanese Pharmacopoeia, 17th Edition (JP XVII), and the pH was adjusted by NaOH (pH 7.4). A total of 0.3 g of 0.4% REB hydrogels was added to the donor side. We collected 50 µL samples of phosphate from the reservoir chamber over time and replaced them with the same volume of 10 mM phosphate buffer. The nanoparticle size distribution, number, and released REB levels in the samples were measured by the NANOSIGHT and HPLC methods described in Section 2.3.

### 2.5. REB Contents in Hamsters Treated with REB Hydrogels

In this study, 10 μM cytochalasin D (phagocytosis inhibitor) [36], 2 μM rottlerin (MP—macropinocytosis inhibitor) [37], 40 μM dynasore (CME—clathrin-dependent endocytosis inhibitor) [38] and 54 μM nystatin (CavME—caveolae-dependent endocytosis inhibitor) [36] were used as inhibitors for each type of endocytosis. A total of 0.1 g of 0.4% REB hydrogel with or without endocytosis inhibitors was applied to the cheek pouch of hamsters and maintained for 2 or 8 h. After that, the hamsters were euthanized under deep isoflurane anesthesia, and the cheek pouches were carefully collected. The collected cheek pouches were homogenized in *N*,*N*-dimethylformamide to extract the REB. Blood was collected from the vena cava, centrifuged at 20,400 g and a temperature of 4 °C for 20 min, and the supernatants were used as samples. REB levels in both the cheek pouch and blood samples were measured by HPLC, as described above.

### 2.6. Measurement of Wound Area in the Hamster Model for Oral Mucositis

Hamsters were anesthetized with isoflurane (3%, rate of flow 1 L/min), and 25 μL of 10% acetic acid was injected into the cheek pouch. After 2 days, the hamsters were used in experiments as a model for oral mucositis. A total of 0.1 g of REB hydrogel was applied once a day (10:00 a.m.) and the wound images were monitored by a digital camera. The wound size was measured daily with an image. The initial areas of the wound (0 days) were as follows: non-treated hamsters (None), 10.8 ± 0.76; vehicle-treated hamsters (Vehicle), 10.2 ± 3.3; R-MPs-treated hamsters, 10.3 ± 3.3; R-NPs-treated hamsters, 10.7 ± 1.9 (mm^2^; means ± standard error of mean (SEM), *n* = 5–8). The values (%) were calculated as the ratio to the initial area of the respective wound.

### 2.7. Measurement of Wound Area in the Hamster Model for Oral Mucositis

The cheek pouches of euthanized hamsters were removed and fixed at room temperature using a tissue quick fixation solution (SUPER FIX, Kurabo Industries, Osaka, Japan). The fixed tissues were prepared in paraffin blocks by the general protocol, and serial sections with a thickness of 4 μm were prepared using a microtome. Hematoxylin and eosin (H&E) staining was performed for morphological observation, and immunostaining was performed with a multi-cytokeratin antibody to identify the oral mucosal epithelium; endogenous peroxidase treatment was performed with 0.3% hydrogen peroxide methanol; and microwave treatment was performed (90 °C, 20 min) in citric acid buffer (pH 6.0) for antigen activation. Samples were incubated with anti-multi-cytokeratin mouse monoclonal antibody (1:200, Clone: AE1/AE3, Leica Biosystems Nussloch GmbH) for 30 min at 37 °C. After three washes with phosphate buffer solution, samples were incubated with universal immune-peroxidase polymer (anti-mouse antibody, Histofine^®^ Simple Stain MAX PO (M), Nichirei Biosciences, Tokyo, Japan) for 30 min at 37 °C. Samples were again washed three times with phosphate buffer solution, color washed with 3,3’-diaminobenzidine tetrahydrochloride (DAB) solution for 30 s, washed with water, and nuclear stained with Meyer’s hematoxylin solution (Muto Chemical Co., Ltd., Tokyo, Japan) for 5 min. Specimens were observed using a biological upright microscope (Power BX-51, Olympus, Tokyo, Japan) with a digital camera (4× and 10× object lenses, DP-71, Olympus), and photographed at the central area of the oral wound.

### 2.8. Statistical Analysis

Data are shown as the mean ± SEM, and ANOVA, Student’s *t*-test and Dunnett’s multiple comparisons were used to analyze statistical differences.

## 3. Results

### 3.1. Design and Characteristics of the R-NPs Gel

**Figure 1:**
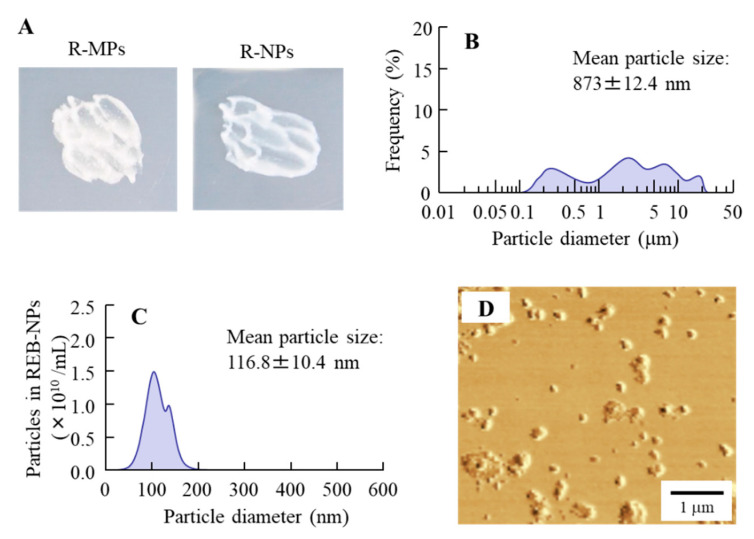
Condition of rebamipide (REB) in REB hydrogels. (**A**) Photographs of the R-MPs gel (hydrogel with incorporated rebamipide microcyrstals) and R-NPs gel (hydrogel with incorporated rebamipide nanocrystals). (**B**,**C**) Particle size in the R-MPs (**B**) and R-NPs (**C**) gels. (**D**) AFM image of the R-NPs gel. Bead mill treatment decreased the particle size of REB to the range of 50–180 nm.

**Figure 2:**
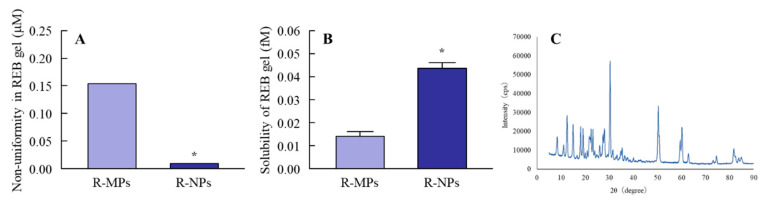
Characteristics of REB in the R-MPs and R-NPs gels. (**A**) Non-uniformity in REB particle distribution in the R-MPs and R-NPs gels. (**B**) Solubility of REB in the R-MPs and R-NPs gels. (**C**) XRD pattern of REB particles after bead mill treatment. *N* = 7. * *p* < 0.05 vs. R-MPs for each category. The mill-treated REB retained its crystal structure, but the uniformity of REB distribution in the R-NPs gel was higher than the non-milled REB in the R-MPs gel. Moreover, solubility of REB was increased by bead mill treatment.

Bead mill is a known method to prepare drug nanocrystals. We attempted to prepare REB nanocrystals using the bead mill method and investigated the characteristics of the hydrogel with REB nanocrystals. Figure 1 shows the particle size distribution of REB in the hydrogel. The aggregates of REB particles were observable in the R-MPs gel (Figure 1A,B) with the naked eye; however, bead mill treatment caused a decrease in REB particle size, after which aggregates were no longer visible in the R-NPs gel (Figure 1A,C). The particle size distribution was 50–180 nm after mill treatment (Figure 1C), and the AFM image also showed the REB particles crushed to nano-size (Figure 1D). In addition, we demonstrated the characteristics of the REB in the R-NPs gel (Figure 2). The REB content in R-NPs gel was uniform in comparison with the R-MPs gel (Figure 2A), and the REB solubility in the R-NPs gel was higher than that in the R-MPs gel (Figure 2B). On the other hand, the crystal form of REB was maintained in the R-NPs gel (Figure 2C), and the crystal structure of REB nanocrystals in the hydrogel was similar to that in the REB microcrystals. These results showed that the form did not affect the difference on its solubility. Moreover, we evaluated the stability of R-NPs gel. In this study, the zeta potential of the REB nanocrystals was −11.9 mV, and no differences were observed in the size, content or form of the REB nanocrystals in hydrogel for one month.

### 3.2. Endocytic Uptake of REB Nanocrystals into Cheek Pouch Tissue

**Figure 3:**
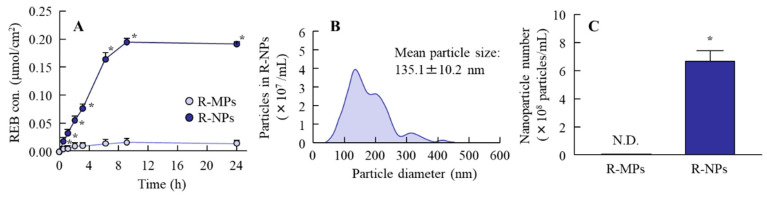
Drug release from R-MPs and R-NPs gels through a 220-nm pore membrane. (**A**) Release behavior of REB from R-MPs and R-NPs gels through a membrane. (**B**) and (**C**) Size distribution (**B**) and number (**C**) of REB nanocrystals in the reservoir chamber 24 h after R-NPs application. *n* = 7. N.D., not detectable. * *p* < 0.05 vs. R-MPs gel for each category. REB was released from the R-NPs gel in the form of nanocrystals.

**Figure 4:**
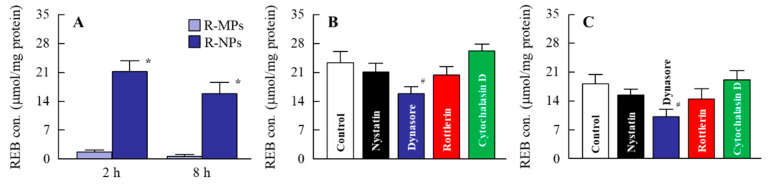
Changes in REB content in the cheek pouch of hamsters treated with REB hydrogels for oral mucositis. (**A**) REB contents in the cheek pouch of hamsters 2 and 8 h after treatment with R-MPs and R-NPs gels. (**B**,**C**) REB contents in the cheek pouch of hamsters treated with endocytosis inhibitors 2 h (**B**) and 8 h (**C**) after the application of R-MPs and R-NPs gels. Control—R-NPs-treated hamster. Nystatin—nystatin-treated hamster treated with R-NPs. Dynasore—dynasore-treated hamster treated with R-NPs. Rottlerin—rottlerin-treated hamster treated with R-NPs. Cytochalasin D—cytochalasin D-treated hamster treated with R-NPs. *n* = 5–7. * *p* < 0.05, vs. R-MPs for each category. ^#^ *p* < 0.05 vs. Control for each category. REB content in hamsters treated with R-NPs gel was higher than in those treated with R-MPs gel; the CME pathway appears to be related to the penetration of REB into the cheek pouch tissues from the hydrogel formulations.

In the investigation of the mechanism for drug permeation in tissues, an evaluation of drug release from the hydrogel is necessary. Figure 3 shows the REB released from the hydrogel. The release of REB was observed for both the R-MPs and R-NPs gels, but the levels released from the R-NPs gel were significantly higher (Figure 3A). Almost all of the REB released from R-MPs gel was of the solution type, while drug nanocrystals were detected in the reservoir chamber after treatment with the R-NPs gel (Figure 3B,C). Next, we examined REB levels in the cheek pouch of hamsters treated with the R-MPs and R-NPs gels (Figure 4A). Eight hours after treatment, the REB levels in hamsters treated with the R-NPs gel were 25-fold higher than in hamsters treated with the R-MPs gel. We then investigated whether endocytosis is related to the uptake of REB into the cheek pouch tissue (Figure 4B,C). Co-treatment with nystatin, rottlerin or cytochalasin D did not affect REB levels in the cheek pouch of hamsters treated with the R-NPs gel. In contrast, co-treatment with dynasore resulted in a significant decrease in tissue REB levels, indicating that CME is related to the uptake of REB into the cheek pouch tissue. We also examined the REB levels in the blood of hamsters 0–8 h after treatment with REB hydrogels. No REB was detected in the plasma of hamsters treated with either the R-MPs or R-NPs gels.

### 3.3. Effect of REB Hydrogel on Oral Wound Healing in the Hamster Model

**Figure 5:**
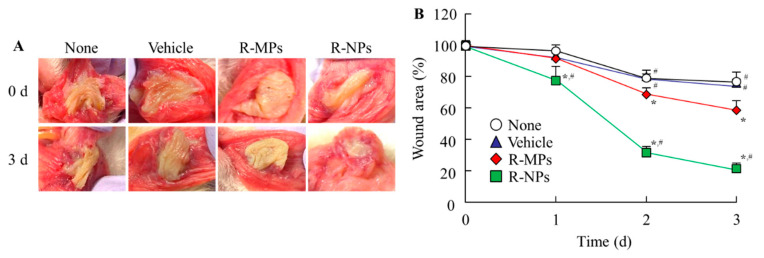
Therapeutic effect of R-NPs gel on wounds in the cheek pouch of hamsters. (**A**) Representative images of the cheek pouch of the hamster model for oral mucositis 0 and 3 d after treatment with REB hydrogels. (**B**), Wound area in the cheek pouch of the hamster model 0–3 d after treatment with REB hydrogels. *n* = 5–8. * *p* < 0.05, vs. Vehicle for each category. ^#^ *p* < 0.05 vs. R-MPs gel for each category. Treatment with REB hydrogel enhanced the therapeutic effect on oral mucositis. The wound areas in hamsters treated with R-NPs gel were significantly smaller than in hamsters treated with R-MPs gel.

**Figure 6:**
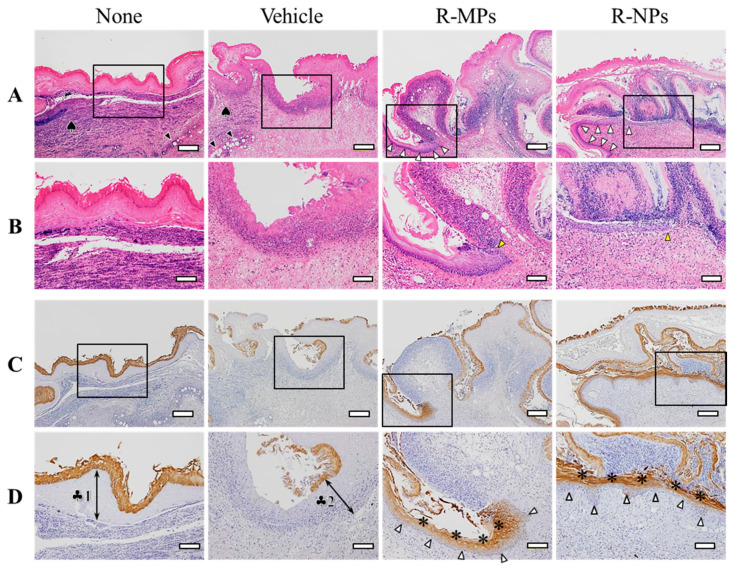
Microscopic effects of the R-NPs gel on oral mucositis. (**A**) Images of H&E-stained cheek pouch tissue specimens from a hamster with oral mucositis three days after treatment with REB hydrogels (4× objective lens; bars indicate 200 μm). (**B**) High magnification images in the areas delineated by the squares in Figure A (10× objective lens; bars indicate 100 μm). (**C**) Images of immunostaining for multi-cytokeratin in the serial sections shown in Figure A (4× objective lens; bars indicate 200 μm). (**D**) High magnification images of the areas delineated by the squares in Figure C (10× objective lens; bars indicate 100 μm).

Figure 5 shows the therapeutic potential of the REB hydrogels for oral mucositis. The wounds in the hamsters injected with acetic acid remained uncured after three days with an area of 8.1 ± 0.5 mm^2^. Although the oral wound in hamsters treated with vehicle also remained uncured after three days (8.0 ± 0.3 mm^2^), hamsters treated with R-MPs gel showed a decrease in the oral wound area to 6.3 ± 0.4 mm^2^. However, treatment with the R-NPs gel significantly enhanced healing of the wound, with the area reduced to 2.3 ± 0.3 mm^2^ three days after the injection of acetic acid. Further, we examined wounds histologically by H&E staining and multi-cytokeratin immunostaining (Figure 6). The None and Vehicle group hamsters showed a degeneration and thickening of the oral mucosal epithelium, migration of inflammatory cells, (♠) and dilated blood vessels (black arrowheads) in the mucosal lamina propria. A regenerated oral mucosal epithelium (white arrowhead) was partially observed in the wounds of hamsters treated with the R-MPs or R-NPs gels, and inflammatory cell levels were reduced in the mucosal lamina propria (Figure 6A). A proliferation of basal cells in the regenerated oral mucosal epithelium was present in hamsters treated with the R-MPs and R-NPs gels (yellow arrowheads, Figure 6B). Oral mucosal epithelial cells containing multi-cytokeratin were stained brown by DAB. The multi-cytokeratin-positive cells in the None group were degenerated and necrotic, and the mucosal lamina propria underlying the mucosal epithelium was thickened (♣1). In the Vehicle group, the degenerated and necrotic mucosal epithelium was almost shedding, and the mucosal lamina propria was thickened as in the None group, with further migration of inflammatory cells (♣2). Regenerated oral mucosal epithelium was observed in the wounds of some of the hamsters treated with the R-MPs or R-NPs gels. The regenerated oral mucosal epithelium included basal cells (white arrowheads) and layers of multi-cytokeratin-positive cells (*) toward the surface of the mucosa (Figure 6C,D).

## 4. Discussion

**Figure 7:**
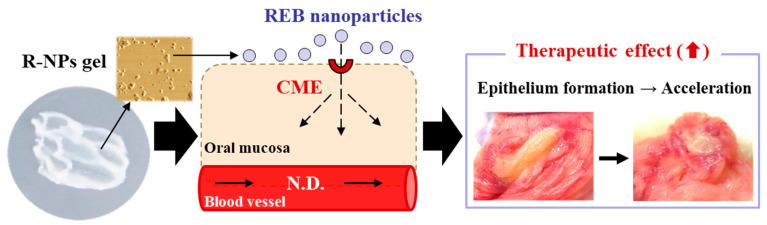
Drug delivery pathway and therapeutic effect of R-NPs gel in the cheek pouch of the hamster model for oral mucositis. N.D., not detectable.

Oral mucositis is the most common painful mucosal lesion, but traditional treatments, such as a mouthwash containing REB, do not provide sufficient treatment or prevention of this condition in serious cases [6,8,39]. Therefore, it is essential to look for effective treatments with few or no adverse effects. In this study, we developed a hydrogel formulation incorporating REB nanocrystals (R-NPs gel) and showed that CME is related to the uptake of REB nanocrystals into the cheek pouch tissue of hamsters. In addition, we found that R-NPs gel releases high levels of REB into tissues and provides a useful therapy for oral mucositis in the hamster model (Figure 7).

Daniel et al. reported limited diffusion of particles larger than 0.5 μm through the mucin layer [31]. Szentkuti [30] showed that particles larger than 1 μm (1.09 μm diameter) do not accumulate on the surface of cell membranes and are undetectable 30 min after application, but the mucoadhesive properties of particles that are 415 nm in diameter are higher and they do accumulate on the apical membranes of surface epithelium cells and remain there for more than 30 min after treatment [30]. These reports suggest that the mucoadhesive properties of a drug increase as particle size decreases, and that the preparation of particles smaller than 400 nm in diameter is needed for application as a drug delivery system (DDS) for therapy of oral mucositis. Break down and build up methods have been used to prepare drug nanocrystals, and we previously succeeded in preparing drug nanocrystals of indomethachin and tranilast with an average particle diameter smaller than 100 nm by applying the bead mill method in the presence of various excipients, such as a cellulose compound and cyclodextrin [40,41]. Moreover, we showed that these drug nanocrystals show high mucoadhesive properties and bioavailability in the small intestine of rats [42,43]. Therefore, we tried to prepare nanocrystals of REB according to our previous protocol [40,41]. The particle size of the REB in the R-NPs gel was in the range of 50–180 nm (Figure 1C,D), so even the largest particle was smaller than 200 nm. Moreover, REB nanocrystals were dispersed uniformly in the hydrogel (Figure 1A) and were released from the R-NPs gel to a significantly greater extent than the R-MPs gel. In addition, REB was released from the hydrogel as drug nanocrystals, since REB nanocrystals were detected in the reservoir chamber of the Franz diffusion cell after passage through a 220 nm pore membrane (Figure 3).

The cheek pouches of a hamster represent a stable environment and drugs that are taken into them do not get washed away. Therefore, wounds in a hamster’s cheek pouch are widely used as an animal model for the study of drug accumulation and the therapeutic effect on oral mucositis. We used this model in our study and found that the accumulation and persistence of REB in the cheek pouch of hamsters treated with the R-NPs gel were remarkably higher in comparison with treatment using the R-MPs gel (Figure 4A). This showed that the cellular uptake of REB nanocrystals was higher than microcrystals in the oral mucosa. In addition, R-NPs gel provided a significant increase in wound healing (Figure 5). The mucosal epithelium of acetic acid-denatured cheek pouch tissues underwent regeneration following treatment with R-MPs and R-NPs, and the number of inflammatory cells observed in the mucosal lamina propria were reduced in comparison with the None and Vehicle treatment groups. Treatment with both the R-MPs and R-NPs gels produced regeneration of the layered structure of the mucosal epithelium, but regeneration was greater in hamsters treated with the R-NPs gel (Figure 6). These results suggest that the REB particle size is suitable for use in a DDS to the oral mucosa, and show that treatment with the R-NPs gel can provide effective therapy for oral wound healing. On the other hand, no REB was detected in the plasma of hamsters treated with either hydrogel. The levels of blood flow and blood volume in the hamster cheek pouch may relate to the non-systemic distribution of REB. These results suggest that the therapeutic effect on oral wound healing by REB is local and not a systemic side effect in the hamster model.

It is important to clarify the mechanism for the enhancement of accumulation, persistence and therapeutic effect of REB in the cheek pouch of hamsters treated with R-NPs gel. In general, solubility is related to tissue penetration and cell uptake, and the solubility of REB in the R-NPs gel was increased by bead mill treatment. Cyclodextrin shows an inclusion ability for drugs, and inclusion enhances the solubility of poorly soluble drugs such as REB. In fact, the solubility of REB in an R-MPs gel without HPβCD is 0.0043 ± 0.0004 fM (*n* = 5), and the liquid REB levels in the hydrogel are lower than in the R-MPs gel containing 5% HPβCD. We also measured REB solubility in the R-NPs gel without 5% HPβCD and found it to be 0.016 ± 0.003 fM (*n* = 5). Thus, the amount of liquid REB is decreased when HPβCD is removed from the R-MPs gel formulation. However, the liquid REB level in the hydrogel formulation incorporating REB nanocrystals and 5% HPβCD was similar to that of the R-MPs gel. These results suggest that the inclusion ability of the drug by HPβCD is enhanced with a decrease in particle size and is related to the increase in REB solubility. Both the liquid (solution) and crystalline types are mixed in the hydrogel formulations incorporating REB nanocrystals, and this enhancement of REB solubility may affect REB release from the hydrogel and uptake into the tissue. Otherwise, the amount of liquid REB in the hydrogel was small, with almost all REB existing as nanocrystals. Therefore, we also investigated the mechanism for the accumulation of drug nanocrystals in the cheek pouch tissue.

The rate of mucosal absorption and retention of drug nanocrystals with a particle diameter smaller than 200 nm is high [29,30,31,32,43], and energy-dependent endocytosis is related to drug uptake in the cells and tissues of the small intestine [42]. Considering this, we investigated whether energy-dependent endocytosis is related to the uptake of REB nanocrystals into cheek pouch tissue by using various inhibitors of energy-dependent endocytosis. Energy-dependent endocytosis is classified into four pathways: Phagocytosis, MP, CME and CavME [44,45]. Large particles (0.5–5 μm) are taken up by phagocytosis, and somewhat smaller particles (100 nm–5 μm) are taken up by MP. CME and CavME relate to the uptake of particles smaller than those taken up by MP: <120 nm for CME and <80 nm for CavME [46]. Each of these energy-dependent endocytosis pathways can be specifically inhibited by different inhibitors: 10 μM cytochalasin D inhibits phagocytosis [36], 2 μM rottlerin inhibits MP [37], 40 μM dynasore inhibits CME [38] and 54 μM nystatin inhibits CavME [36]. Co-treatment of R-NPs gel and either cytochalasin D, rottlerin or nystatin had no effect of the amount of REB taken up by cheek pouch tissue. REB content in the cheek pouch of hamsters co-treated with R-NPs gel and dynasore were significantly decreased (Figure 4B,C). These results suggest that the REB nanocrystals are taken up into cheek pouch tissue by the CME pathway, resulting in the enhancement of wound healing (Figure 5 and Figure 6).

## 5. Conclusions

We designed a hydrogel formulation incorporating REB nanocrystals (R-NPs gel) and found that most of the REB is released from the hydrogel as drug nanocrystals, which are taken up into the tissue through the CME pathway. REB provides a persistent effect, resulting in an enhancement of oral wound healing. It is possible that the R-NPs gel can provide useful therapy for serious oral mucositis. The development of formulations incorporating drug nanocrystals, such as hydrogels and mouthwashes, may be helpful when designing oral DDS for poorly soluble drugs.
